# A Case Report of Pearson Syndrome: Addressing the Complexities beyond Anemia

**DOI:** 10.1159/msy/adrag020

**Published:** 2026-09-24

**Authors:** Niladri Das, Haseena Sait, Anshima Singh, Rajesh Kumar Maurya, Vishakha Gupta, Kausik Mandal

**Affiliations:** a Department of Medical Genetics, Sanjay Gandhi Postgraduate Institute of Medical Sciences, Lucknow, Uttar Pradesh, India; b Department of Pathology, Sanjay Gandhi Postgraduate Institute of Medical Sciences, Lucknow, Uttar Pradesh, India

**Keywords:** Pearson syndrome, Mitochondrial DNA deletion, Sideroblastic anemia, Exocrine pancreatic insufficiency, Lactic acidosis

## Abstract

**Introduction:**

Pearson syndrome (PS) is a mitochondrial deletion disorder typically presenting as transfusion-dependent sideroblastic anemia with varying involvement of different cell lines, along with multisystem involvement during infancy. This fatal condition requires a high degree of clinical suspicion due to its phenotypic heterogeneity.

**Case Presentation:**

We present the case of an 8-month-old infant with sideroblastic anemia and thrombocytopenia, initially showing a negative whole exome sequencing (WES) result from peripheral blood. Bone marrow reexamination revealed additional findings, including vacuolization of erythroid and myeloid precursors. With clinical suspicion of PS, analysis of the mitochondrial genome in WES data identified a novel 4.9-kb mitochondrial DNA deletion (m.8482_13477del4996) covering the *COX3, ND3, ND4, ND5, ATP6*, and *ATP8* genes, with subsequent multisystem involvement confirming the diagnosis.

**Conclusion:**

This case underscores the importance of integrating laboratory findings with genetic testing, as focused analysis of the mitochondrial genome in cases of sideroblastic anemia in bone marrow can give a definitive diagnosis of PS.

**Established Facts**

1. Pearson syndrome (PS) presents with severe hypoproliferative anemia in early infancy, multiorgan dysfunction, lactic acidosis, pancreatic insufficiency, renal tubulopathy, failure to thrive, endocrine disorders, ringed sideroblasts, and vacuolization of erythroid and myeloid precursor cells in bone marrow.
2. PS is a rare mitochondrial cytopathy caused by single large-scale mitochondrial DNA deletions, most commonly by a de novo 4.977-kb deletion [m.8470_13446del4977 (NC_012920.1)].

**Novel Insights**

1. We identified a novel 4.9-kb mitochondrial DNA deletion (m.8482_13477del4996) covering the COX3, ND3, ND4, ND5, ATP6, and ATP8 genes on mitochondrial genome analysis in whole exome sequencing (WES) data, thus highlighting the importance of such analysis when WES yields a negative result.

## Introduction

Pearson syndrome (PS) is a rare mitochondrial cytopathy that falls within the phenotypic spectrum of disorders caused by single large-scale mitochondrial DNA deletions (SLSMDs). The incidence of PS is unknown, with around 150 patients described in the literature [1]. The disorder typically manifests with severe hypoproliferative anemia in early infancy, followed by progressive symptoms and multiorgan dysfunction, including lactic acidosis, pancreatic insufficiency, renal tubulopathy, failure to thrive, and endocrine disorders [2, 3]. The presence of ringed sideroblasts and marked vacuolization of erythroid and myeloid precursor cells on bone marrow examination is a clue for the diagnosis [3, 4]. When there is strong clinical suspicion of PS, testing for large mitochondrial DNA deletions using long PCR and/or Southern blot is required instead of performing whole exome sequencing (WES). Due to diverse phenotypic manifestations and a lack of awareness among clinicians, there is often a significant delay in diagnosis. While in some patients, spontaneous resolution of anemia occurs, the prognosis is usually poor, with the majority dying during early childhood [5].

We report an 8-month-old infant who presented with transfusion-dependent sideroblastic anemia. Initial WES from peripheral blood was reported to be negative. Bone marrow reexamination revealed ringed sideroblasts with vacuolization of marrow precursors. Analysis of the mitochondrial genome in WES data led to the identification of a 4.9-Kb deletion of mitochondrial DNA, and subsequent multisystemic involvement confirmed the diagnosis of PS. This case highlights the complementary nature of laboratory findings and genetic testing, where bone marrow examination provided critical clues that, alongside genetic reanalysis, led to an accurate diagnosis.

## Case Report

This case report was prepared in accordance with the CARE reporting guidelines. The completed CARE checklist is provided as online supplementary material (for all online suppl. material, see https://doi.org/10.1159/msy/adrag020). An 8-month-old male infant presented with progressive pallor, requiring multiple transfusions since 3 months of age. He was born at term to non-consanguineous parents via normal vaginal delivery, with a birth weight of 3,000 g. There was no significant family history. On examination, the infant had severe pallor, but his anthropometry was appropriate for age, with a weight of 8 kg (−1.25 Z score), length of 70 cm (−1.45 Z score), and head circumference of 45 cm (−0.33 Z score) according to IAP 2015 growth charts. A systemic examination revealed soft hepatomegaly (liver span 7 cm) without splenomegaly, and other systemic examinations were normal. Initial investigations showed normocytic normochromic anemia and thrombocytopenia. Serologies for parvovirus B19 and cytomegalovirus were negative. Bone marrow aspiration, which was performed due to undiagnosed persistent anemia and thrombocytopenia, revealed 12–15% ring sideroblasts, suggestive of sideroblastic anemia. WES done on genomic DNA extracted from peripheral blood of the child did not identify any pathogenic variants associated with sideroblastic anemia. The child was not put on any antibiotic prophylaxis at discharge and continued to receive monthly transfusions of packed RBCs for anemia.

At 9 months of age, the infant was admitted to our department with an acute onset of fever and cough. His cardiovascular and respiratory exams were normal; hepatomegaly was present (liver span 10 cm). Laboratory tests showed mild anemia and thrombocytopenia (Hb: 10.2 g/dL, total leukocyte count: 4,900/μL, absolute neutrophil count: 1,715/μL, platelets: 38,000/μL). Chest radiography showed right lower lobe opacity, and a 2D echocardiography was normal. He was treated symptomatically with intravenous fluids, antipyretics, and intravenous ceftriaxone. Due to persistent fever and pneumonia, oral azithromycin was added.

During the hospital stay, the child developed decompensated shock and hypoglycemia (RBS: 40 mg/dL), which was managed with oxygen, IV fluids, inotropes, and IV dextrose. Blood gas analysis revealed high anion gap metabolic acidosis with elevated lactate levels (pH: 7.232, HCO_3_: 10.5 mmol/L, PCO_2_: 25.8 mm Hg, lactate: 63 mg/dL). Further tests revealed febrile neutropenia (total leukocyte count: 1,600/μL, absolute neutrophil count: 500/μL) and elevated inflammatory markers (serum C-reactive protein and procalcitonin), suggesting sepsis. Following this, antibiotics were upgraded to intravenous meropenem, teicoplanin, and polymyxin B. Blood cultures failed to reveal any significant growth. Persistent anemia and thrombocytopenia required transfusions of packed RBCs and platelets. The deranged coagulation profile (prothrombin time of 19.6 s, INR 1.4, and aPTT of 118.9 s) was corrected with fresh frozen plasma. Following these interventions, the child stabilized, and inotropes were discontinued after 24 h.

Given the persistently elevated lactate levels even after stabilization, a mitochondrial disorder was suspected. A re-evaluation of the bone marrow aspiration slides revealed prominent cytoplasmic vacuolization in erythroid and myeloid precursors in addition to 15% ring sideroblasts (Fig. 1). Reanalysis of WES data focusing on the mitochondrial genome was done using the MitoSeek tool, which is an open-source bioinformatics pipeline capable of extracting and analyzing mtDNA from WES data without requiring dedicated mitochondrial sequencing. Using this tool, mtDNA reads were extracted directly from aligned sequencing data (BAM files), and reads confidently assigned to the mitochondrial genome were preferentially used to minimize interference from nuclear mitochondrial DNA sequences. We identified a novel 4.9-kb deletion in the mitochondrial DNA region (m.8482_13477del4996) covering the *COX3*, *ND3*, *ND4*, *ND5*, *ATP6*, and *ATP8* genes, confirming the diagnosis of PS [6] (Fig. 2).

**Fig. 1.**
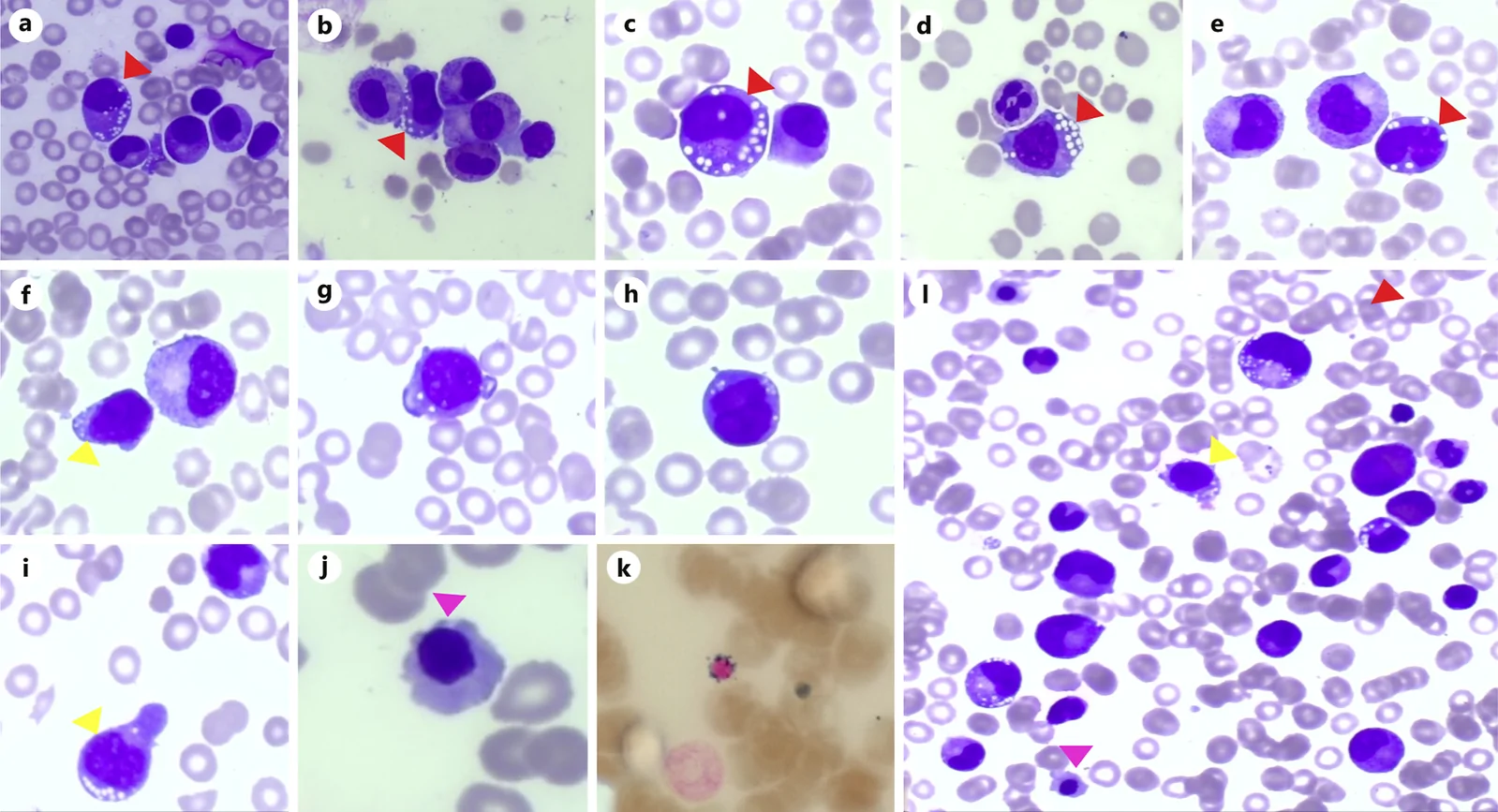
Bone marrow features of Pearson syndrome (Wright-Giemsa stain). **a–e** Vacuoles seen in the myeloid lineage cells (marked with a red arrowhead). **f–i** Vacuoles seen in the erythroid lineage cells (marked with a yellow arrowhead). **j** Increased cytoplasm in the late normoblast with abnormal hemoglobinization (marked with a red arrowhead). **k** Ringed sideroblasts (iron staining). **l** All above features (marked with respective arrowheads).

**Fig. 2.**
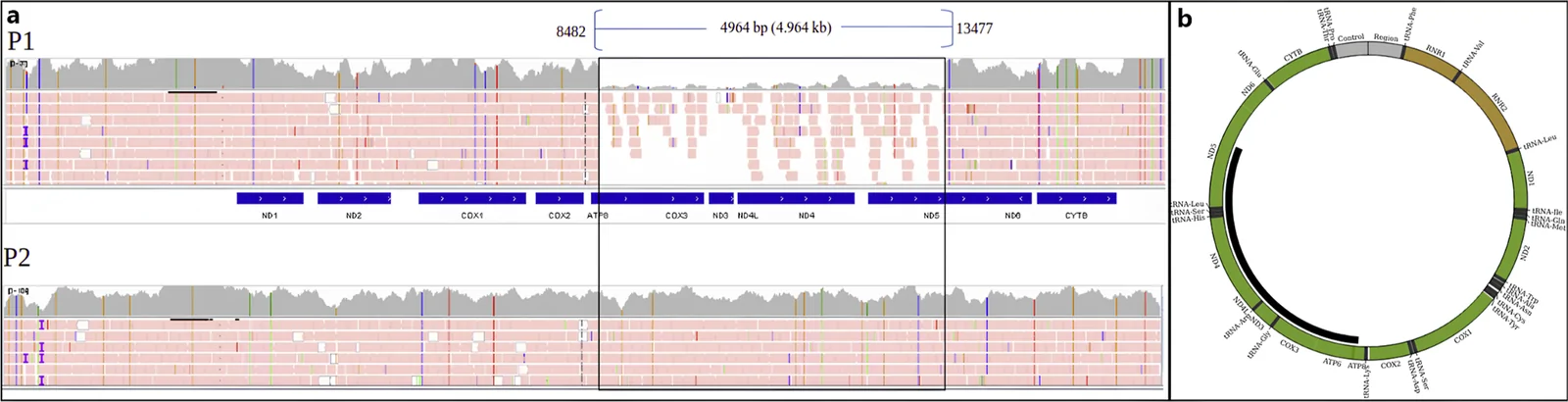
**a** Mitochondrial gene visualization by the Integrated Genome Viewer (IGV) tool. P1 (our patient) when compared to P2 (control) shows poor coverage of mt region (8,482–13,477) suggesting 4.964-kb deletion. **b** Circular mtDNA plot specifying the location of the deleted region (black bar) consisting of the genes *ND5*, *tRNA*- (*Leu*, *Ser*, *His*, *Arg*, and *Gly*), *ND4*, *ND4L*, *ND3*, *COX3*, *ATP6* and *ATP8*. Devised with the help of the MitoBreak database.

Reverse phenotyping was then conducted, revealing markedly increased stool fat indicative of exocrine pancreatic insufficiency. There was also increased serum lipase, which can be attributed to the underlying sepsis. Further evaluation revealed polyuria, and elevated urinary electrolytes and protein levels confirmed proximal tubulopathy. The relevant investigations are summarized in Table 1.

**Table 1.** Laboratory parameters of the patient

Investigations	Patient’s value (normal range)			
complete blood count	D1	D14	D23	D29
Hb, g/dL	10.2 (14–17.5)	4.3	7.9	12.6
TLC, per µL	4,900 (4,000–10,000)	1,600	1,200	500
ANC, per µL	1,715 (2,500–6,000)	560	360	75
Platelet count, per µL	38,000 (150,000–450,000)	6,000	6,000	6,000
Kidney function tests and serum electrolytes (D14)				
BUN/urea, mg/dL	7.18/15.5 (5.9–20)/(12.8–42.8)			
Creatinine, mg/dL	0.3 (0.7–1.5)			
Sodium, mmol/L	132 (135–145)			
Potassium, mmol/L	3.0 (3.4–4.5)			
Calcium (total/ionized), mg/dL	8.4/5 (9–11)/(4.8–5.6)			
Phosphorus, mg/dL	2 (4.0–7.0)			
Magnesium, mg/dL	2 (1.7–2.4)			
Chloride, mmol/L	108 (98.0–107.0)			
Urine analytes and electrolytes (D26)				
Urine osmolality, mosmol/kg	160 (500–850)			
Urinary protein, mg/dL	31.9 (0–10)			
Urine creatinine, mg/dL	5 (20–320)			
Urine glucose and ketones	Negative			
Urine phosphorus, mg/dL	20.70 (0.85–1.0)			
Urine uric acid, mg/dL	8.40 (0.3–0.5)			
Urine calcium creatinine ratio, mg/g creatinine	1,780 (12–244)			
Urine chloride, mmol/L	46.80 (120–250)			
Urine sodium, mmol/L	95.8 (>20)			
Urine potassium, mmol/L	18.78 (20–40)			
Liver function tests and coagulation profile (D14)				
Total/direct bilirubin, mg/dL	0.52/0.32 (0.5–1.2)/(0.1–0.4)			
Total protein/albumin, g/dL	3.76/1.99 (6.4–8.3)/(3.5–5.2)			
AST/ALT/ALP/LDH, U/L	36/51/115/213 (5–40)/(8–41)/(30–115)/(207–414)			
PT-INR	PT: 19.6 s (ctrl 13.5 s), INR 1.4			
aPTT, s	118.9 (29.1)			
Fibrinogen, mg/dL	210 (50–250)			
Metabolic workup (D14)				
Serum ammonia, µmol/L	22 (10–54)			
Serum lactate, mg/dL	98.7 (4.5–19.8)			
TMS	Normal			
Lipid profile (D14)				
Total cholesterol, mg/dL	58 (<200)			
Triglyceride, mg/dL	232 (<150)			
HDL, mg/dL	12 (>40)			
VLDL, mg/dL	46.4 (2–30)			
LDL, mg/dL	4 (<100)			
Pancreatic parameters				
Stool fat (droplets/hpf) (D19)	>50 (<50)			
Serum amylase/lipase (D28), U/L	12/534 (22–80)/(≤38)			
BMA slides review (D14)				
Trilineage hematopoiesis with reduced erythroid precursors with cytoplasmic vacuolization in myeloid as well as erythroid lineage cells and ringed sideroblasts (<15%)				
Inflammatory markers (D14)				
CRP, mg/dL	119 (up to 5)			
Procalcitonin, ng/mL	27.93 (<0.05, ≥10 suggests severe sepsis)			
Serum ferritin, µg/L	780 (30–400)			
Cultures and infectious workup				
Blood cultures (D3, D10, D19)	Sterile			
Blood cultures (D28)	*Klebsiella pneumoniae* (multidrug-resistant)			
Urine culture	Sterile			
Serum galactomannan (D19) (units)	0.15 (≤0.5)			

D, day of admission; Hb, hemoglobin; TLC, total leukocyte count; ANC, absolute neutrophil count; BUN, blood urea nitrogen; HDL, high-density lipoprotein; VLDL, very low-density lipoprotein; LDL, low-density lipoprotein; AST, aspartate aminotransferase; ALT, alanine aminotransferase; ALP, alkaline phosphatase; LDH, lactate dehydrogenase; PT, prothrombin time; INR, international normalized ratio; aPTT, activated partial thromboplastin time; CRP, C-reactive protein.

The persistent pancytopenia and febrile neutropenia were empirically managed with antibiotics (intravenous meropenem, teicoplanin, and polymyxin B), with the addition of antifungal therapy (amphotericin B). The patient required repeated transfusions of packed RBCs and platelets due to persistent anemia and thrombocytopenia. Lactic acidosis was managed with intermittent sodium bicarbonate. Electrolyte imbalances were corrected. Calcitriol was administered for proximal tubulopathy. A mitochondrial cocktail, including biotin, coenzyme Q, carnitine, thiamine, riboflavin, and folic acid, was also initiated. Granulocyte colony-stimulating factor (G-CSF) injection could not be initiated for myelosuppression due to financial constraints.

The child developed a new right middle lobe patch on chest radiography 27 days after admission. Repeat blood cultures revealed the growth of multidrug-resistant *Klebsiella pneumoniae*. Despite aggressive management, the pancytopenia persisted, and ANC showed a further decline. Unfortunately, the child ultimately succumbed to septic shock as a result of the complications of his multisystemic illness 1 month after admission.

## Discussion

We present the case of an infant who had an initial diagnosis of transfusion-dependent sideroblastic anemia without multisystem involvement. The management of this patient’s pancytopenia, sepsis, and intermittent metabolic crises during hospital admission proved challenging, particularly due to the absence of a clear underlying etiology. A thorough bone marrow evaluation revealed a crucial diagnostic clue: marked vacuolation of myeloid and erythroid precursors. This finding, alongside the sideroblastic anemia, raised the suspicion of PS. Thus, we proceeded with exome reanalysis and analyzed the mitochondrial genome in WES data, which led to the identification of a 4.9-kb deletion in the mitochondrial DNA. The deletion was initially missed on WES as the mitochondrial genome was not analyzed in WES at that time.

PS is a congenital multisystem disorder that typically presents within the first year of life. Clinical features often include severe anemia with neutropenia and/or thrombocytopenia, ring sideroblasts in the bone marrow, pancreatic exocrine insufficiency, and lactic acidosis. The disorder is usually fatal in infancy due to infections related to neutropenia or refractory lactic acidosis [5]. Some survivors may later develop Kearns-Sayre syndrome, characterized by ophthalmoplegia, ataxia, retinitis pigmentosa, conduction defects, and myopathy [4]. Shwachman-Diamond syndrome clinically presents similarly to PS in infancy with exocrine pancreatic insufficiency and cytopenias. Bone marrow is, however, hypocellular in Shwachman-Diamond syndrome, while individuals with PS have normal bone marrow cellularity, ring sideroblasts, and vacuolization of bone marrow progenitors, pancreatic fibrosis rather than lipomatosis, and absence of bone lesions [7]. Ringed sideroblasts and marked vacuolation of hematopoietic precursors can also be observed in copper deficiency and myelodysplastic syndromes. Our case had clinical and laboratory features similar to previously reported cases. The genetic confirmation of PS in our case elucidated the previously masked multiorgan abnormalities, which had been obscured by the sepsis presentation.

Management of PS is quite challenging. As infections can trigger life-threatening metabolic decompensations, the prevention of infections by vaccinations and treatment with early antipyretic and antibiotic therapy is needed [1]. However, the role of prophylactic antimicrobial therapy in PS is not established. For those who survive infancy, a complete recovery of bone marrow and pancreatic function may be achieved with appropriate supportive care, including packed cell transfusions, treatment of metabolic acidosis, and G-CSFs [7]. G-CSF may correct neutropenia, but erythropoietin and eltrombopag have limited effect on anemia and thrombocytopenia [1]. Hematopoietic stem cell transplantation can address the hematological manifestations of PS [8]. There is insufficient evidence regarding its utility in prolonging survival and correcting or preventing the progressive multiorgan dysfunction in these patients. Hematopoietic stem cell transplantation was attempted in 6 patients with PS, and none were successful due to treatment-related complications [1]. Moreover, its utility is questioned due to the high rate of spontaneous resolution of anemia in many patients. The recent development of novel pharmacological and non-pharmacological therapies, including cell and gene therapies for mitochondrial diseases, has opened exciting avenues and holds potential as a therapeutic option for PS in the near future [9–11]. Mitochondrial augmentation therapy was tried for PS, in which CD34+ hematopoietic progenitors were harvested from patients and augmented with maternally derived healthy mitochondria and then infused intravenously. This study showed some promising results and laid the foundation for future clinical trials of mitochondrial augmentation therapy for the treatment of patients with mtDNA disorders [9].

PS is relatively homogeneous, with one-third of cases involving a 4.977-kb deletion [m.8470_13446del4977 (NC_012920.1)] in mitochondrial DNA, typically occurring de novo in the mother’s oocytes during germline development or in the embryo during embryogenesis. [2, 3]. However, various other types of deletions in mtDNA are also reported in PS [12–14]. Methods used for identifying these deletions include targeted techniques like Southern blot, long-range PCR, quantitative PCR, and droplet digital PCR (ddPCR), and non-targeted techniques like next-generation sequencing [3]. Analysis of SLSMDs in next-generation sequencing-based techniques using bioinformatics tools like the MitoSeek tool is a highly sensitive method that can precisely identify the deletion breakpoints. However, it is more complex, time-consuming, and expensive than other techniques and does not provide highly accurate quantification of heteroplasmy in SLSMDs because deletion-supporting reads are more difficult to quantify accurately from short-read sequencing [3, 6]. Southern blot was once considered the gold standard for detecting SLSMDs but has now been replaced by molecular methods because it is labor-intensive, requires larger amounts of high-quality DNA, is less sensitive in detecting low-level heteroplasmy, and may fail to differentiate an SLSMD from multiple mtDNA deletions in the same region. Long-range PCR is a simpler and faster technique for detection of SLSMDs but cannot quantify heteroplasmy levels. Quantitative PCR methods such as ddPCR are more expensive but are considered the gold standard for targeted quantification, as it has the highest sensitivity and provide absolute quantification of heteroplasmy [3].

Our case had a novel 4.9-kb deletion in the mitochondrial DNA region (m.8482_13477del4996), which is highly similar to the common 4,977-bp deletion and affects the same mitochondrial genes. As the mother is clinically unaffected, the empiric risk of recurrence in future pregnancies is very low (at or below 1%). However, there is a rare possibility that a mother harbors the deletion at a very low heteroplasmic level and transmits it to her offspring. However, even if the deletion is inherited, prenatal testing for subsequent pregnancies is not predictive of specific outcomes due to potential variability in the mutated DNA inherited by the offspring [3].

Our study had several limitations. Validation of the deletion and heteroplasmy quantification by an alternative method like ddPCR and analysis of the SLSMD in tissues other than peripheral blood could have provided stronger evidence but could not be done in our case due to financial constraints. Performing maternal testing across various tissues (e.g., blood, urine, buccal swabs, and saliva) in our case would have definitively confirmed whether the deletion was de novo. However, maternal testing could not be done as the family did not consent to testing of the mother during the period of hospital admission.

To conclude, this case highlights the importance of mitochondrial genome analysis in WES data when WES yields a negative result, as most of the currently used exome capture kits include probes capable of capturing mtDNA. It also highlights the complexity of diagnosing and managing PS, a rare congenital disorder with a broad spectrum of clinical manifestations and multiorgan involvement. The confirmation of PS provided critical insights into the patient’s overall clinical picture, revealing previously masked multiorgan abnormalities.

## Acknowledgments

The authors would like to thank the parents of the child for their support.

## Statement of Ethics

The study has been granted an exemption from requiring ethics approval by the Institutional Ethics Committee, Sanjay Gandhi Postgraduate Institute of Medical Sciences, Lucknow, Uttar Pradesh, India. Written informed consent was obtained from the parents of the child for publication of the details of their medical case and any accompanying images.

## Conflict of Interest Statement

The authors have no conflicts of interest to declare.

## Funding Sources

This study was not supported by any sponsor or funder.

## Author Contributions

Conception and design: H.S. and K.M.; methodology: H.S., K.M., and N.D.; acquisition of data: N.D., H.S., K.M., A.S., and V.G.; bioinformatic analysis: R.K.M. and H.S.; writing – original draft: N.D., H.S., K.M., A.S., and V.G.; and writing – review and editing: N.D., H.S., K.M., and R.K.M. K.M. will act as guarantor for the manuscript. The manuscript has been read and approved by all the authors, and the requirements for authorship have been met. Each author believes that the manuscript represents honest work.

## Data Availability

All data supporting the findings of this study are available within the paper and its online supplementary material. Further inquiries can be directed to the corresponding author.
